# Supplementary material for: Allosteric regulation and crystallographic fragment screening of SARS-CoV-2 NSP15 endoribonuclease
Source: Nucleic Acids Res. 2023 Apr 28;51(10):5255–70. doi: 10.1093/nar/gkad314 (PMC10250223; doi:10.1093/nar/gkad314)
Supplement: gkad314_Supplemental_Files [file gkad314_supplemental_files.zip › Supplementary datasets.zip!/224/Refine_9/01-BUSTER/Cycle-1/shell.01/R_Rfree.html]

# Rxpct factors in resolution bins at cycle 0


|  |  |  |  |  |  |  |  |  |
| --- | --- | --- | --- | --- | --- | --- | --- | --- |
|  | **<d> (Å)**  Rxpct\_Work | Rxpct\_Free | Rxpct\_All | |  |  |  |  |
|  | 15.0574 | 0.3511 | 0.4251 | 0.3537 |  |
|  | 8.6934 | 0.2198 | 0.2651 | 0.2224 |  |
|  | 6.7339 | 0.2351 | 0.2379 | 0.2353 |  |
|  | 5.6912 | 0.2137 | 0.2751 | 0.2157 |  |
|  | 5.0191 | 0.1530 | 0.2076 | 0.1563 |  |
|  | 4.5400 | 0.1290 | 0.1761 | 0.1314 |  |
|  | 4.1762 | 0.1402 | 0.1736 | 0.1421 |  |
|  | 3.8878 | 0.1481 | 0.1917 | 0.1500 |  |
|  | 3.6520 | 0.1622 | 0.2028 | 0.1642 |  |
|  | 3.4544 | 0.1632 | 0.2073 | 0.1658 |  |
|  | 3.2858 | 0.1682 | 0.2167 | 0.1710 |  |
|  | 3.1397 | 0.1748 | 0.2510 | 0.1781 |  |
|  | 3.0115 | 0.1885 | 0.2150 | 0.1897 |  |
|  | 2.8978 | 0.2110 | 0.2026 | 0.2105 |  |
|  | 2.7961 | 0.2136 | 0.2201 | 0.2139 |  |
|  | 2.7044 | 0.2231 | 0.2309 | 0.2235 |  |
|  | 2.6212 | 0.2237 | 0.2512 | 0.2253 |  |
|  | 2.5452 | 0.2432 | 0.2504 | 0.2437 |  |
|  | 2.4754 | 0.2648 | 0.3251 | 0.2683 |  |
|  | 2.4111 | 0.2682 | 0.2453 | 0.2672 |  |
|  | **Overall** | 0.1878 | 0.2231 | 0.1896 |  |

# Rxpct factors in resolution bins at cycle 1


|  |  |  |  |  |  |  |  |  |
| --- | --- | --- | --- | --- | --- | --- | --- | --- |
|  | **<d> (Å)**  Rxpct\_Work | Rxpct\_Free | Rxpct\_All | |  |  |  |  |
|  | 15.0574 | 0.3439 | 0.4133 | 0.3463 |  |
|  | 8.6934 | 0.2142 | 0.2622 | 0.2170 |  |
|  | 6.7339 | 0.2278 | 0.2355 | 0.2283 |  |
|  | 5.6912 | 0.2082 | 0.2687 | 0.2102 |  |
|  | 5.0191 | 0.1530 | 0.2082 | 0.1564 |  |
|  | 4.5400 | 0.1311 | 0.1733 | 0.1333 |  |
|  | 4.1762 | 0.1405 | 0.1738 | 0.1424 |  |
|  | 3.8878 | 0.1497 | 0.1930 | 0.1517 |  |
|  | 3.6520 | 0.1639 | 0.2060 | 0.1659 |  |
|  | 3.4544 | 0.1616 | 0.2038 | 0.1641 |  |
|  | 3.2858 | 0.1688 | 0.2149 | 0.1715 |  |
|  | 3.1397 | 0.1743 | 0.2499 | 0.1776 |  |
|  | 3.0115 | 0.1888 | 0.2105 | 0.1897 |  |
|  | 2.8978 | 0.2106 | 0.1983 | 0.2100 |  |
|  | 2.7961 | 0.2146 | 0.2177 | 0.2147 |  |
|  | 2.7044 | 0.2235 | 0.2388 | 0.2243 |  |
|  | 2.6212 | 0.2237 | 0.2536 | 0.2255 |  |
|  | 2.5452 | 0.2445 | 0.2424 | 0.2444 |  |
|  | 2.4754 | 0.2706 | 0.3322 | 0.2742 |  |
|  | 2.4111 | 0.2723 | 0.2340 | 0.2706 |  |
|  | **Overall** | 0.1871 | 0.2216 | 0.1888 |  |

# Rxpct factors in resolution bins at cycle 2


|  |  |  |  |  |  |  |  |  |
| --- | --- | --- | --- | --- | --- | --- | --- | --- |
|  | **<d> (Å)**  Rxpct\_Work | Rxpct\_Free | Rxpct\_All | |  |  |  |  |
|  | 15.0574 | 0.3358 | 0.4041 | 0.3382 |  |
|  | 8.6934 | 0.2070 | 0.2550 | 0.2098 |  |
|  | 6.7339 | 0.2154 | 0.2286 | 0.2162 |  |
|  | 5.6912 | 0.2000 | 0.2607 | 0.2020 |  |
|  | 5.0191 | 0.1496 | 0.2090 | 0.1532 |  |
|  | 4.5400 | 0.1275 | 0.1665 | 0.1295 |  |
|  | 4.1762 | 0.1372 | 0.1713 | 0.1391 |  |
|  | 3.8878 | 0.1468 | 0.1846 | 0.1485 |  |
|  | 3.6520 | 0.1621 | 0.2048 | 0.1641 |  |
|  | 3.4544 | 0.1606 | 0.1990 | 0.1628 |  |
|  | 3.2858 | 0.1677 | 0.2081 | 0.1700 |  |
|  | 3.1397 | 0.1734 | 0.2464 | 0.1766 |  |
|  | 3.0115 | 0.1869 | 0.2153 | 0.1882 |  |
|  | 2.8978 | 0.2053 | 0.1948 | 0.2048 |  |
|  | 2.7961 | 0.2089 | 0.2202 | 0.2094 |  |
|  | 2.7044 | 0.2196 | 0.2388 | 0.2206 |  |
|  | 2.6212 | 0.2233 | 0.2500 | 0.2249 |  |
|  | 2.5452 | 0.2407 | 0.2531 | 0.2414 |  |
|  | 2.4754 | 0.2600 | 0.3341 | 0.2643 |  |
|  | 2.4111 | 0.2732 | 0.2439 | 0.2720 |  |
|  | **Overall** | 0.1830 | 0.2182 | 0.1848 |  |

# Rxpct factors in resolution bins at cycle 3


|  |  |  |  |  |  |  |  |  |
| --- | --- | --- | --- | --- | --- | --- | --- | --- |
|  | **<d> (Å)**  Rxpct\_Work | Rxpct\_Free | Rxpct\_All | |  |  |  |  |
|  | 15.0574 | 0.3244 | 0.3827 | 0.3264 |  |
|  | 8.6934 | 0.1938 | 0.2443 | 0.1967 |  |
|  | 6.7339 | 0.1945 | 0.2501 | 0.1977 |  |
|  | 5.6912 | 0.1775 | 0.2441 | 0.1796 |  |
|  | 5.0191 | 0.1395 | 0.1990 | 0.1431 |  |
|  | 4.5400 | 0.1249 | 0.1593 | 0.1267 |  |
|  | 4.1762 | 0.1361 | 0.1779 | 0.1384 |  |
|  | 3.8878 | 0.1482 | 0.1987 | 0.1505 |  |
|  | 3.6520 | 0.1599 | 0.2093 | 0.1623 |  |
|  | 3.4544 | 0.1577 | 0.2113 | 0.1608 |  |
|  | 3.2858 | 0.1664 | 0.2098 | 0.1689 |  |
|  | 3.1397 | 0.1734 | 0.2342 | 0.1761 |  |
|  | 3.0115 | 0.1858 | 0.2181 | 0.1872 |  |
|  | 2.8978 | 0.2019 | 0.2097 | 0.2023 |  |
|  | 2.7961 | 0.2053 | 0.2292 | 0.2064 |  |
|  | 2.7044 | 0.2167 | 0.2375 | 0.2178 |  |
|  | 2.6212 | 0.2206 | 0.2574 | 0.2228 |  |
|  | 2.5452 | 0.2375 | 0.2635 | 0.2390 |  |
|  | 2.4754 | 0.2608 | 0.3216 | 0.2643 |  |
|  | 2.4111 | 0.2774 | 0.2655 | 0.2769 |  |
|  | **Overall** | 0.1776 | 0.2199 | 0.1798 |  |
